# Eukaryotic translation elongation factor-1 alpha is associated with a specific subset of mRNAs in *Trypanosoma cruzi*

**DOI:** 10.1186/s12866-015-0436-2

**Published:** 2015-05-19

**Authors:** Lysangela Ronalte Alves, Camila Oliveira, Samuel Goldenberg

**Affiliations:** Instituto Carlos Chagas, Fiocruz – PR, Curitiba, Parana Brazil

**Keywords:** *Trypanosoma cruzi*, Gene expression, mRNPs, EF-1 alpha, Non-canonical function

## Abstract

**Background:**

Regulation of gene expression in trypanosomatids is mainly posttranscriptional. Tight regulation of mRNA stability and access to polysomes allows *Trypanosoma cruzi* to adapt to different environmental conditions during its life cycle. Posttranscriptional regulation requires association between mRNAs and specific proteins to form mRNP complexes. Proteins that lack a canonical RNA-binding domain, such as eukaryotic elongation factor-1α (EF-1α), may also associate with mRNPs. EF-1α is conserved in many organisms, and it plays roles in many cellular processes other than translation, including RNA transport, the cell cycle, and apoptosis.

**Results:**

In a previous study, EF-1α was found associated with mRNP-forming mRNAs in polysome-free fractions both in epimastigotes growing under normal conditions and in nutritionally stressed parasites. This finding suggested the possibility that EF-1α has a non-canonical function. Thus, we investigated the dynamics of EF-1α in association with *T. cruzi* epimastigote mRNAs under normal and stressed nutritional conditions. EF-1α is expressed throughout the parasite life cycle, but it shows a slight decrease in protein levels in the metacyclic trypomastigote form. The protein is cytoplasmically localized with a granular pattern in all forms analyzed. Following puromycin treatment, EF-1α migrated with the heaviest gradient fractions in a sucrose polysome profile, indicating that its association with large protein complexes was independent of the translation machinery. We next characterized the EF-1α-associated mRNAs in unstressed and stressed epimastigotes. We observed that specific subsets of mRNAs were associated with EF-1α-mRNPs in unstressed or stressed epimastigotes. Some mRNAs were identified in both physiological conditions, whereas others were condition-specific. Gene ontology analysis identified enrichment of gene sets involved in single-organism metabolic processes, amino acid metabolic processes, ATP and metal ion binding, glycolysis, glutamine metabolic processes, and cobalt and iron ion binding.

**Conclusion:**

These results indicate that in *T. cruzi*, as in other eukaryotes, EF-1α may play a non-canonical cellular role. We observed the enrichment of functionally related transcripts bound to EF-1α in normal growth conditions as well as in nutritionally stressed cell indicating a potential role of EF-1α mRNP in stress response.

**Electronic supplementary material:**

The online version of this article (doi:10.1186/s12866-015-0436-2) contains supplementary material, which is available to authorized users.

## Background

The life cycle of *Trypanosoma cruzi* involves two intermediary hosts and at least three well-defined developmental stages that are based on morphology and biological characteristics [1, 2]. The metacyclogenesis process, in which replicating epimastigotes transform into infective and non-replicating metacyclic trypomastigotes, occurs in the midgut of the invertebrate triatomine host and can be mimicked in vitro under chemically defined conditions [3, 4]. Interestingly, the differentiation of epimastigotes is triggered by nutritional stress, and different sets of genes are expressed in normally growing epimastigotes and in nutritionally stressed epimastigotes [5].

The mechanisms of gene expression regulation in *T. cruzi* present some peculiarities. In the absence of typical RNA polymerase II promoter regions, transcription begins within the divergent strand-switch region and occurs in both directions. As a result, dozens to hundreds of genes with unrelated functions are transcribed in the same polycistronic unit, which upon processing results in different levels of protein production [6, 7]. These features demonstrate that the regulation of gene expression in these organisms is mainly post-transcriptional and is based on mechanisms involving the localization, translation and degradation of mRNAs [6, 8, 9]. In recent years, several mechanisms have been described as possible strategies for gene expression regulation.

The translation is a very important step of gene expression regulation. The ribosome profiling was developed in order to have a better view of the mRNAs that are actually engaged to translation and how is the relevance of the translational control [10]. For *Trypanosoma brucei* and *Plasmodium falciparum*, it has been shown that translation has a very important role in gene expression regulation in these parasites [11–13]. Another mechanism is the organization of mRNAs into post-transcriptional operons or regulons, where mRNAs with related functions are associated with similar sets of proteins in mRNPs, thereby enabling their coordinated expression [5, 14–17]. Another possible mechanism is the association of RNAs into RNA granules [18–20] that ultimately determine the fate of mRNAs within the cell [5, 14–17].

Elongation factor 1α (EF-1α) binds GTP, and the GTP-bound form interacts with aminoacyl-tRNA and carries it to the A site of the ribosome [21]. Control of EF-1α protein levels is important for normal cell function, as EF-1α acts as a regulator of the cell cycle. Accordingly, EF-1α overexpression is associated with many tumor types [22]. EF-1α has also been described as an actin-binding protein in several species [23, 24]. In addition, other non-canonical functions have been assigned to EF-1α because of its involvement in various cellular processes, including metabolism [25], cytoskeletal organization [26], oncogenic transformation [27, 28], apoptosis and anti-apoptosis [29, 30], and gene expression [31]. It has also been demonstrated that EF-1α plays a role in connecting the translation machinery with the cytoskeleton, thus permitting mRNA localization in chicken embryo fibroblasts. EF-1α associates with F-actin; the resulting complex binds to beta-actin mRNA and anchors it in the cellular protrusions of the fibroblast [31]. EF-1α is also found in RNA granules, which are transported by microtubules in neurons [32].

In *T. cruzi*, EF-1α [23] was previously shown to bind mRNAs not only in polysomes but also in polysome-free fractions, both in exponentially growing epimastigotes and in parasites under nutritional stress [33]. The association of EF-1α with mRNA complexes that are not associated with the protein translation apparatus permitted us to better characterize EF-1α mRNPs by identifying their associated transcripts. This approach yielded further insights into the role of EF-1α mRNPs in the regulation of *T. cruzi* gene expression.

## Results and discussion

### *T. cruzi* EF-1α genomic organization and protein characteristics

**Fig. 1:**
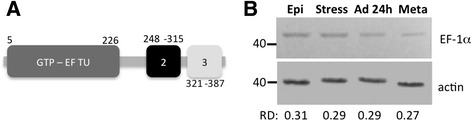
Scheme of the EF-1α protein from *T. cruzi*. **(a)** The domains are indicated; the GTP-binding domain is dark grey, the second domain is black and the third domain is light grey. The numbers indicate the positions of the domains within the protein. **(b)** Western-blot analysis of *T. cruzi* extracts with antisera against EF-1α and actin. Epi: exponentially growing normal epimastigotes. Stress: epimastigotes under nutritional stress. Ad 24 h: epimastigotes adhered to the substrate. Meta: metacyclic trypomastigotes. Ten μg of protein was loaded into each lane. Top panel: anti-EF-1α antibody signal (1:300). Bottom panel: anti-β-actin used for normalization (1:500). The relative densities of the bands (RD) are indicated. The molecular mass standards are indicated (kDa)

*T. cruzi* EF-1α is organized as a multicopy gene family that includes 11 tandem copies on chromosome 5 [34]. The EF-1α protein has three domains. Domain I is responsible for GTP binding, domain II is responsible for recognizing the 3' end of the tRNA, and domain III connects the aminoacyl-tRNA to the ribosome (Fig. 1a) [35]. The *T. cruzi* EF-1α protein has an estimated molecular weight of 43 kDa, and it is highly conserved among trypanosomes and other eukaryotes, with 95 % identity when compared to trypanosomes and 76 % identity when compared to *Homo sapiens.*

**Fig. 2:**
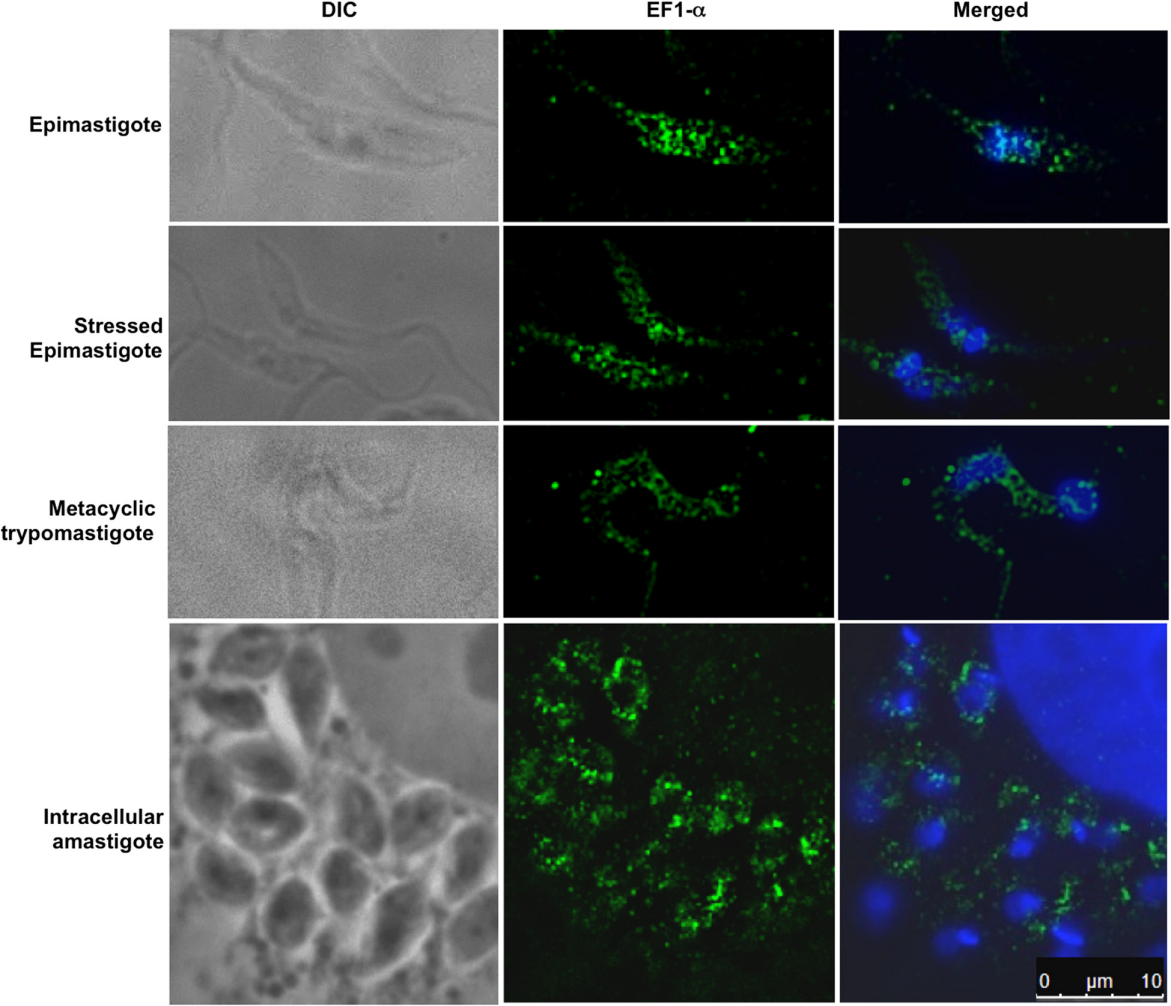
Immunolocalization assay. Cells were incubated with anti-EF-1α antibody (1:300). Immune complexes were detected by reaction with Alexa 488-labelled goat anti-rabbit antibody (1:400). Kinetoplasts and nuclei were stained with DAPI, and the obtained image was merged with the EF-1α image. Bar = 10 μm

EF-1α protein expression is controlled throughout the parasite life cycle (Fig. 1b). Accordingly, proteomic data have shown that EF-1α protein levels decrease in metacyclic trypomastigotes compared to epimastigotes [36]. All analyzed forms of EF-1α showed a granular distribution throughout the cytoplasm of the parasite, with a decrease of signal in metacyclic trypomastigotes that corroborated the proteomic and western blot results (Fig. 2). The immunofluorescence controls are shown in the Additional file 4: Figure S1.

### EF-1α is present in large mRNP complexes that are not associated with translation machinery

**Fig. 3:**
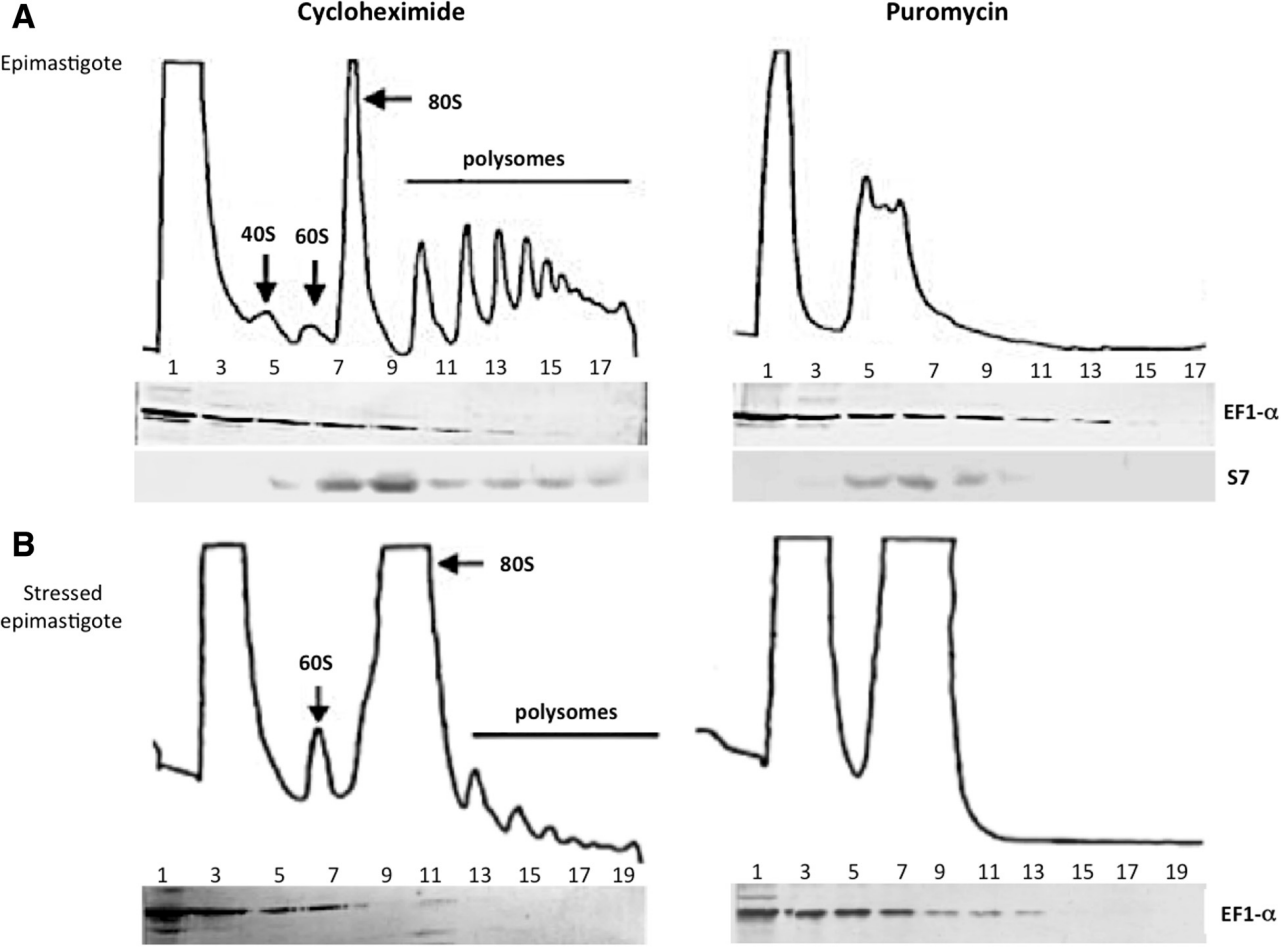
Polysome fractionation. **(a)** Unstressed epimastigote polysome profile. **(b)** Stressed epimastigote polysome profile. Fractions were analyzed by western blotting with antiserum against EF-1α (1:300 dilution) and S7 ribosomal protein (1:500). The numbers in the fractions are related to: 1 and 3 – free fraction, 5 – 40 S, 7 – 60 S, 9-17 monosomes to heavy polysomes

To investigate the distribution of the EF-1α protein in complexes, we analyzed its sedimentation profile in sucrose density gradients (15–55 %). Protein extracts of fractions from unstressed and stressed epimastigotes treated with cycloheximide or puromycin were analyzed by western blot using antisera against the EF-1α protein and the ribosomal protein S7 as a control (Fig. 3).

A typical polysome sedimentation profile was obtained from epimastigotes treated with cycloheximide, a drug that inhibits the function of the translation factor eEF2. eEF2 acts in the translocation of peptidyl-tRNA from the A site to the P site of the ribosome. Thus, cycloheximide treatment blocks translation elongation and maintains the association between ribosomes and mRNA. The EF-1α protein was detected in light fractions and in fractions corresponding to as many as 3 ribosomes (Fig. 3a). Alternatively, for polysome dissociation analysis, parasites were treated with puromycin, a drug that causes the premature release of the polypeptide chain from the ribosome. Interestingly, even after polysome dissociation by puromycin, the distribution of EF-1α in the gradient was unchanged. EF-1α migrated with the heaviest fractions under these conditions, indicating a translation machinery-independent association with large complexes (Fig. 3a).

Nutritional stress is a trigger for *T. cruzi* differentiation [3, 4]. In nutritionally stressed epimastigotes treated with cycloheximide, the polysome profile was characteristic of stressed cells. The polysomes were smaller and less abundant than in normal culture conditions. EF-1α protein was also present over a range of lighter to heavier fractions, but much less EF-1α was detected in the heavier fractions in stressed conditions than in unstressed epimastigotes, a finding that reflects the lower translational activity in stressed conditions (Fig. 3). The EF-1α migration pattern in stressed parasites after puromycin treatment was similar to that of unstressed puromycin-treated epimastigotes (Fig. 3b).

The results of the polysome sedimentation analysis suggested that, in addition to its association with ribosomes during translation, EF-1α is associated with heavier complexes that co-sediment with polysomes but are not necessarily associated with the translation machinery. This type of profile is typical of RNA granules that consist of mRNP complexes that are not involved in translation. One example is the mRNP complex involving the *T. cruzi* protein TcDHH1, which forms large complexes associated with mRNAs that co-sediment with polysomes but that are connected to the storage or degradation of associated transcripts [37]. EF-1α has a possible non-canonical function that very likely involves the formation of non-translatable mRNP complexes.

### mRNAs associated with EF-1α mRNPs

Next, we aimed to investigate whether EF-1α was involved in mRNP complexes and to characterize the associated mRNAs in unstressed and nutritionally stressed epimastigotes. The ribonomic assay was performed with biological triplicates and was followed by RNA-seq. The EF-1α immunoprecipitation was confirmed by western blot showing the protein in the elution fraction (Additional file 5: Figure S2). Rabbit pre-immune serum under the same conditions was used as a control. The sequencing data were deposited in the NCBI Sequence Read Archive (SRA) database under accession number SRP051179. We used an RPKM (Reads Per Kilo base per Million) value of at least 50, an FDR (False Discovery Rate) ≤ 0.01 and a minimum of a four-fold change with respect to the control (pre-immune serum) as a threshold for selecting mRNAs that were associated with EF-1α-containing mRNPs. Using these criteria we identified 85 transcripts that were enriched for EF-1α mRNP by at least fourfold over the control (anti-EF-1α vs. pre-immune control in epimastigotes extracts) (Additional file 1: Table S1). In stressed parasites, 133 mRNAs were identified as enriched in mRNPs in comparison to the control (anti-EF-1α vs. pre-immune control in stressed epimastigotes extracts) (Additional file 2: Table S2). The RNA-seq data was validated by qRT-PCR (Additional file 6: Figure S3).

**Fig. 4:**
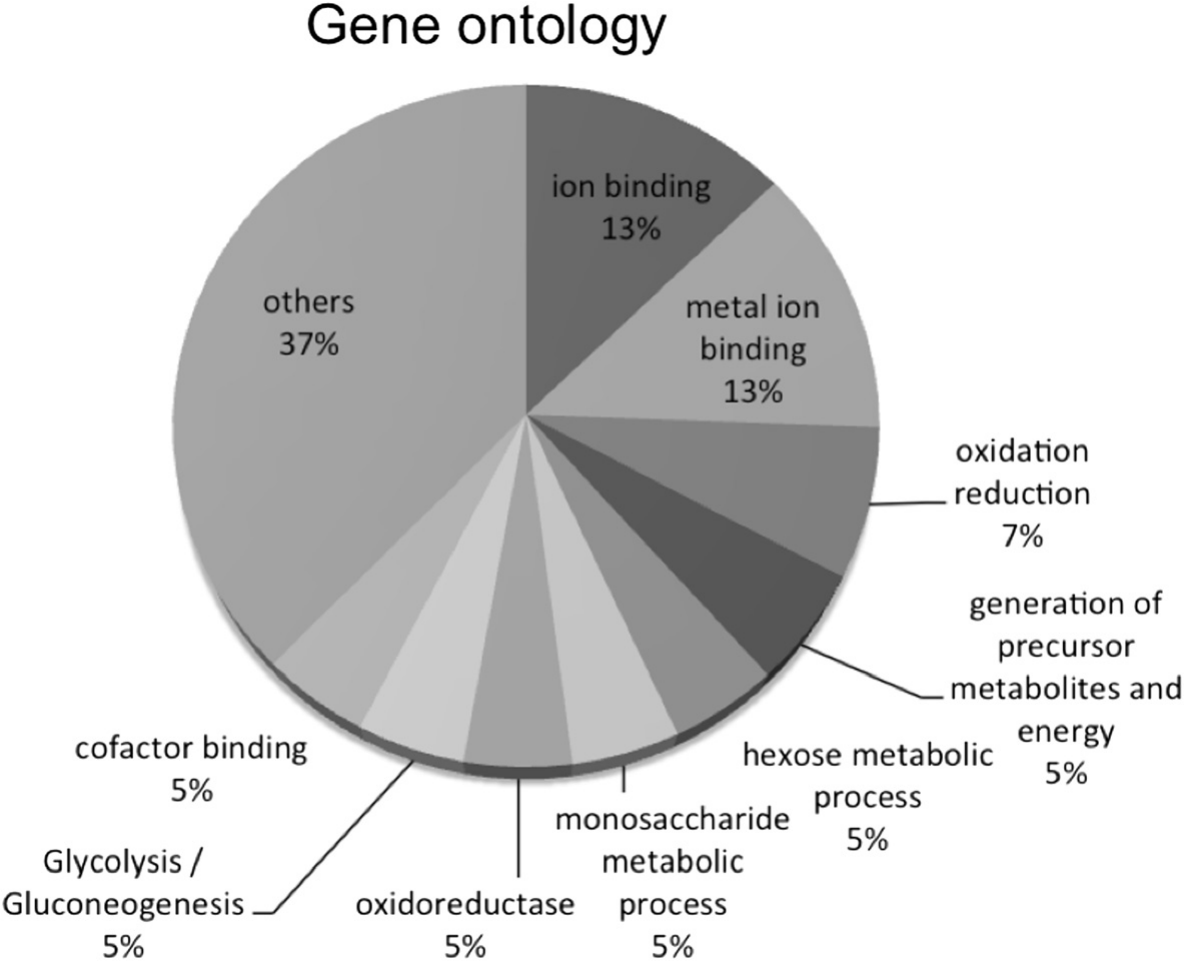
Gene Ontology pie chart. The percentage indicates the enrichment for the GO terms in the transcripts associated to EF-1α in epimastigotes and stressed epimastigotes

**Fig. 5:**
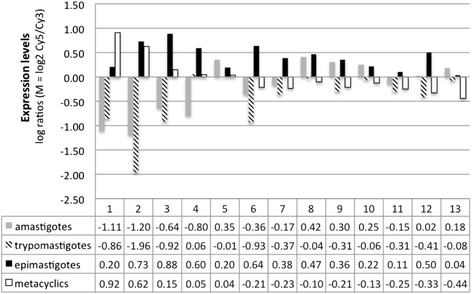
mRNA expression levels of the *T. cruzi* life cycle stages (trypomastigotes, amastigotes, epimastigotes, and metacyclic trypomastigotes) determined by microarray analysis [45]. Six hybridizations were performed and consisted of three dye-swap experiments from three independent samples (biological replicates). In each case, the experimental sample was from a single life-cycle stage and the control sample was an equal mixture of all four life-cycle stages [45]. Expression values for 2-channel microarray experiments are shown as log ratios (M = log_2_ Cy5/Cy3) [45]. The numbers refer to different mRNAs: 1- TcCLB.510879.130 (hypothetical protein); 2- TcCLB.503975.50 (hypothetical protein); 3- TcCLB.509599.130 (serine/threonine protein kinase); 4- TcCLB.504163.60 (hypothetical protein); 5- TcCLB.509455.114 (hypothetical protein); 6- TcCLB.504005.54 (hypothetical protein); 7- TcCLB.508719.70 (flagellum targeting protein Kharon 1); 8- TcCLB.511365.80 (mitochondrial carrier protein); 9- TcCLB.506825.200 (hypothetical protein); 10- TcCLB.506529.324 (hypothetical protein); 11- TcCLB.506529.508 (glucose-6-phosphate isomerase); 12- TcCLB.510535.100 (cysteine C peptidase CPC); 13- TcCLB.511801.60 (mitochondrial DEAD-box helicase)

We observed specific subsets of mRNAs that were associated with EF-1α mRNPs in unstressed or stressed epimastigotes. Some transcripts were identified in both physiological conditions, while others were specific to a given condition. A possible functional correlation among the enriched transcripts was investigated using the Gene Ontology (GO) categories of the DAVID functional annotation and Blast2GO tools [38, 39] (Fig. 4 and Table 1). Upon grouping the mRNAs on the basis of the GO terms, we noticed the enrichment of terms specific to biological processes in epimastigotes and stressed epimastigotes. In unstressed epimastigotes, there was a slight enrichment in terms related to ATP binding and metal ion binding (Fig. 4 and Table 1). However, for nutritionally stressed epimastigotes, it was possible to identify a greater number of GO categories, such as single-organism metabolic processes, amino acid metabolic processes, glycolysis, glutamine metabolic processes, cobalt ion binding and iron ion binding (Fig. 4 and Table 1). The glycolysis pathway in trypanosomatids is very similar to other eukaryotes, with the exception of two main differences: some enzymes in the pathway are compartmentalized within the glycosome, and inhibitory control of hexokinase and phosphofructokinase is absent [40–42]. The compartmentalization of the enzymes seems to be important for parasite survival [43, 44]. In *T. brucei*, expression of phosphoglycerate kinase and triose phosphate isomerase in the cytoplasm rather than in the glycosome leads to parasite death [43, 44]. Interestingly, the mRNAs that encode the glycolytic enzymes enclosed in glycosomes were found to associate with EF-1α. Enolase, fructose-biphosphate aldolase, glucose-6-phosphate isomerase, aldolase-1 epimerase, hexokinase and alcohol dehydrogenase transcripts were also found associated with EF-1α-containing mRNPs (Additional file 1: Table S1 and Additional file 2: Table S2). It is tempting to speculate that EF-1α associates with a subset of transcripts to transport them to the vicinity of glycosomes. Such transport would allow localized translation and more efficient transport into the organelle, hence minimizing the number of active enzymes in the cytoplasm. We also analyzed the expression patterns of some transcripts that were associated with EF-1α by using the transcriptome data of the *T. cruzi* life cycle available at tritrypdb.org [45] (Fig. 5). An observable pattern of expression was shared by all the transcripts; namely, they are upregulated in epimastigotes (as in the case of glucose-6-phosphate isomerase, isocitrate dehydrogenase and lanosterol 14-alpha-demethylase), and they all encode enzymes associated with carbohydrate and lipid metabolism (Fig. 5).

**Table 1:** Gene ontology terms for EF-1α associated transcripts

Gene ontology	Count	Enrichment score	*p*-value	Benjamini
**Epimastigote**				
Nucleotide binding	8	0.83	1.00E-01	9.80E-01
Kinase	5	0.83	1.50E-01	9.70E-01
ATP binding	6	0.56	1.90E-01	9.80E-01
Transition metal ion binding	3	0.1	7.20E-01	9.90E-01
**Stressed epimastigote**				
Generation of precursor metabolites and energy	7	2.84	4.30E-02	3.00E-04
Monosaccharide metabolic process	6	2.84	4.50E-02	2.50E-03
Glutamine family amino acid metabolic process	4	1.59	5.20E-02	1.10E-03
Proteolysis	7	1.07	9.70E-01	3.10E-01

The RNA regulon theory, which states that mRNAs encoding proteins with related functions are associated with specific mRNP complexes [6, 16, 17] could explain the results obtained for EF-1α in *T. cruzi*, however further experiments need to be performed in order to validate this hypothesis for EF-1α. One example of a regulon in *T. cruzi* is the TcZC3H39-mRNP, which binds a specific subset of mRNAs that encode the cytochrome oxidase C complex. Interestingly, this association occurs in response to stress [5]. A comparison of transcripts associated with EF-1α and with TcZC3H39 show no target overlap, indicating that the transcripts associated with each protein form distinct mRNPs in the cell.

The stress triggers a global rearrangement of the metabolism in the cell, including the translation machinery. Some RNAs shift from the polysomes to RNA granules as p-bodies or stress granules while others remain associated to polysomes and are actively translated, for example heat shock proteins and chaperones, as a translational reprogramming necessary in order to rapidly respond to the stress condition [19, 20]. The shifts observed on the targets associated to EF1-α protein suggest that this protein might play a non-canonical role in *T.cruzi* through participation in distinct mRNP complexes, such as its potential role in localized translation in epimastigotes or translation repression in parasites under nutritional stress conditions (Additional file 1: Table S1, Additional file 2: Table S2 and Additional file 6: Figure S3).

The mRNA and protein levels in a cell are not necessarily correlated. Recent data using polysome-profiling techniques allow a more precise quantification of the mRNAs actively engaged in translation [46–48]. The transcripts stability is not sufficient to explain the level of the encoded proteins since this is a reflex of translation efficiency. However, the role of RNA binding proteins associated to mRNP complexes cannot be underestimated since they ultimately define the fate of a given mRNA as being translated, stored or degraded.

## Conclusions

Specific subsets of mRNAs were enriched in EF-1α-containing mRNPs, indicating a non-canonical role for this protein in *T. cruzi*. We are aware that EF-1α is a rather abundant and positively charged cytoplasmic protein that might bind nonspecifically to mRNAs. However, the experimental conditions used in the previous work (isolation of polysomal and non-polysomal mRNPs with 300 mM NaCl) minimize spurious and non-specific binding between proteins and mRNAs [33]. Sucrose gradient sedimentation analysis showed that EF-1α migrated to the heavier fractions of the gradient irrespective of whether polysomes were dissociated with puromycin, indicating its association with large mRNP complexes. The mRNAs bound to EF-1α mRNPs were enriched for metabolic processes; notably, six of these transcripts encoded glycosomal proteins that are involved in the glycolysis pathway. This result indicates that in *T. cruzi*, as well as in other eukaryotes, EF-1α might have additional functions other than in translation. The results presented here allow us to hypothesize that EF-1α acts by associating with transcripts that must be localized to specific cellular sites for translation and during the stress response might be acting in mRNPs that associates to translation repression. Our findings shed new light on the understanding of post-transcriptional regulation in *T. cruzi*.

## Methods

### EF-1α cloning and antibody production

The complete coding sequence of the *EF-1α* gene (TcCLB.510119.9 and TcCLB508949.4) was amplified by PCR using the forward primer ATGGGGAAGGACAAGGTG and the reverse primer GGACTTGATCGACTTGGG. The amplicon was cloned into the pDNOR vector from the Gateway platform. The gene was then sub-cloned into the pDEST15 expression vector to allow recombinant His-tagged protein purification. The recombinant protein was expressed in an *Escherichia coli* DH5α p*LysS* strain and purified using an electron affinity column. The purified recombinant protein was then inoculated into rabbits to obtain polyclonal antibodies [3].

### Parasite cultures

*T. cruzi* Dm 28c epimastigotes were cultured in liver infusion tryptose (LIT) medium at 28 °C. Epimastigotes in the late exponential growth phase were obtained from five-day cultures (density of 5 × 10^7^ parasites ml^−1^). For stress conditions, epimastigotes from five-day cultures were harvested by centrifugation at 7,000 × g for 5 min at 10 °C and then incubated for 2 h at 28 °C in TAU medium (190 mM NaCl, 17 mM KCl, 2 mM MgCl_2_, 2 mM CaCl_2_, 8 mM phosphate buffer, pH 6.0) at a density of 5 × 10^8^ parasites ml^−1^. The assay for the parasite life cycle was conducted as previously described [3, 4].

### Western blot analysis

For western blot analysis, *T. cruzi* extracts were separated by SDS-PAGE on a 15 % polyacrylamide gel; the separated bands were then transferred to a nitrocellulose membrane. Nonspecific binding sites were blocked by incubating the membrane with 5 % non-fat milk powder and 0.1 % Tween 20 in PBS for 30 min. The membrane was then incubated for 1 h with specific antibodies against EF-1α diluted at 1:500, thoroughly washed in PBS, and incubated with goat alkaline phosphatase-conjugated anti-rabbit IgG (Sigma) diluted at 1:10,000. The color reaction was developed using 5-bromo-4-chloro-3-indozyl phosphate and nitroblue tetrazolium (Promega). Quantification of bands was performed using ImageJ software (Bethesda, MD, USA).

### Immunofluorescence and imaging

Immunofluorescence assays were conducted as previously described [46]. Image capture and deconvolution were performed using the Leica AF6000 Modular System with LAS AF 3.x.

### Sucrose density gradient separation

*T. cruzi* polysomes were isolated on sucrose gradients. Cells (1 × 10^9^) were incubated with 100 μg ml^−1^ cycloheximide for 10 min or with 2 mM puromycin for one hour. They were kept on ice for 5 min before being pelleted by centrifugation (7,000 × g for 5 min at 4 °C) and washed with cold TKM buffer (10 mM Tris, pH 7.4, 300 mM KCl and 10 mM MgCl_2_). The cell pellet was resuspended in 900 μl TKM supplemented with 10 μg ml^−1^ heparin, 10 μM E-64, and 1:100 EDTA-free protease cocktail (Roche). The suspension was transferred to a new tube containing 100 μl of lysis buffer (TKM supplemented with 10 % (v/v) NP-40 and 2 M sucrose), and homogenized by repeated passages through a pipette. Lysis was monitored using phase-contrast microscopy. The lysate was centrifuged at 18,000 × g at 4 °C for 5 min. The cleared supernatant (500 μl; equivalent to 5 × 10^8^ cells) was layered onto linear 15-to-55 % sucrose density gradients prepared in TKM buffer supplemented with inhibitors (10 μM E-64, 1 mM PMSF, and 1 mg ml^−1^ heparin) and centrifuged at 4 °C for 2 h at 365,000 × g in a Beckman SW41 rotor. After centrifugation, 500-μl fractions were collected using the ISCO gradient fractionation system.

### EF-1α immunoprecipitation RNA-seq

To identify mRNAs associated with EF-1α protein or complexes, rabbit anti-EF-1α antibody (15 μl) was incubated with 50 μl of goat anti-rabbit magnetic beads (New England Biolabs) and 40 U/ml RNAse OUT (Invitrogen) for 16 h at 4 °C with shaking. Cytoplasmic extract corresponding to 1 × 10^9^ cells was then incubated with the beads. The beads were then washed three times with IMP1 buffer. The immunoprecipitated RNAs were eluted and purified with the RNeasy® (Qiagen) kit by using the “Animal Cells I” protocol in the manufacturer’s manual but with an additional column-based DNase treatment step. The purified RNAs were then subjected to deep sequencing using the SOLiD 3 plus platform. The experiments were performed in triplicate and the rabbit pre-immune serum was used as a control in the same conditions as the immune serum.

### *In silico* data analysis

The sequencing data obtained were analyzed using the CLC Genomics Workbench© v 5.5.1. The reads were trimmed on the basis of quality using a threshold phred score of 15. The *T. cruzi* reference genome used for mapping was obtained from the NCBI database (AAHK01), and the alignment was performed with the following parameters: additional upstream and downstream sequences of 100 bases; minimum number of reads, 10; maximum number of mismatches, 2; nonspecific match limit, −2; use of colorspace encoding. The minimum similarity of the reads mapped to the reference genome was 70 %. We selected possible targets of EF-1α mRNPs by using the beta binomial statistical test [47], with a p-value-corrected FDR of ≤ 1 % and a minimum four-fold change with respect to the control (pre-immune serum) as the threshold for significance.

### Functional annotation

To conduct the gene ontology term enrichment analysis used for the classification of transcripts and to conduct Kegg pathway map analysis we used the DAVID annotation tool (david.abcc.ncifcrf.gov/home.jsp) [48] and Blast2GO [38]. For enrichment analysis and functional annotation, the statistical p-value was based on the EASE score, a modified one-tailed Fisher’s exact test p-value. An EASE value of 0 represents perfect enrichment, while values of up to 0.05 were considered to indicate strong enrichment in the annotation categories considered.

## Additional files

Additional file 1:
**RNAs associated to EF-1a in epimastigotes.**
Additional file 2:
**RNAs associated to EF-1a in stressed epimastigotes.**
Additional file 3:
**Supplemental methods.**
Additional file 4:
**Controls of the immunofluorescence assay.**
Additional file 5:
**EF-1a immunoprecipitation western-blot.**
Additional file 6:
**Validation of the RNA-seq by qRT-PCR.**
Additional file 7:
**Primers used in this study.**
