# Supplementary material for: Eukaryotic translation elongation factor-1 alpha is associated with a specific subset of mRNAs in Trypanosoma cruzi
Source: BMC Microbiol. 2015 May 19;15:104. doi: 10.1186/s12866-015-0436-2 (PMC4436862; doi:10.1186/s12866-015-0436-2)
Supplement: Additional file 1: — RNAs associated to EF-1a in epimastigotes. [file 12866_2015_436_MOESM1_ESM.pdf]

Feature ID	Product	Baggerley's test: EPI EF1a vs CT EF1a normalized values - Weighted proportions fold change	Baggerley's test: EPI EF1a vs CT EF1a normalized values - Bonferroni	Baggerley's test: EPI EF1a vs CT EF1a normalized values - FDR p-value correction	EPI EF1a - Normalized means	CT EF1a - Normalized means
TcCLB.510785.10	hypothetical protein	-10.639	0.00%	0.00%	380.168	35.732
TcCLB.507951.215	hypothetical protein, conserved	-5.165	0.00%	0.00%	315.504	61.090
TcCLB.508681.31	hypothetical protein	-6.976	0.00%	0.00%	282.086	40.437
TcCLB.508233.60	heat shock protein 100 (clp protein), putative	-5.168	1.23%	0.00%	224.143	43.369
TcCLB.511211.120	activated protein kinase C receptor, putative,guanine nucleotide-binding protein beta subunit-like protein	-4.117	0.63%	0.00%	219.967	53.430
TcCLB.479517.30	hypothetical protein	-7.190	0.17%	0.00%	212.758	29.591
TcCLB.510093.10	ATPase, putative	∞	0.00%	0.00%	166.40	0
TcCLB.511869.9	dynein heavy chain, putative	-4.538	0.03%	0.00%	157.21	34.643
TcCLB.511233.59	hypothetical protein, conserved	∞	0.00%	0.00%	138.25	0
TcCLB.506825.200	hypothetical protein	-6.986	0.00%	0.00%	136.40	19.524
TcCLB.506401.12	protein kinase domain, putative	∞	0.00%	0.00%	119.40	0
TcCLB.508409.9	26S proteasome regulatory non-ATPase subunit, putative	-5.920	0.01%	0.00%	119.13	20.123
TcCLB.510759.200	hypothetical protein, conserved	-9.929	0.00%	0.00%	117.77	11.861
TcCLB.509901.160	hypothetical protein, conserved	-4.306	0.00%	0.00%	114.84	26.673
TcCLB.510889.80	serine/threonine protein phosphatase, putative	-5.672	0.00%	0.00%	104.62	18.446
TcCLB.508873.210	mucin-associated surface protein (MASP), putative	∞	0.00%	0.00%	103.02	0
TcCLB.506247.190	nuclear lim interactor-interacting factor, putative	∞	0.45%	0.00%	98.54	0
TcCLB.505997.21	N-acetyltransferase complex ARD1 subunit (pseudogene), putative	∞	0.00%	0.00%	98.01	0
TcCLB.503415.10	hypothetical protein (pseudogene)	-9.215	0.00%	0.00%	94.50	10.255
TcCLB.511021.60	hypothetical protein, conserved	-7.431	0.00%	0.00%	94.24	12.682
TcCLB.510439.61	heat shock 70 kDa protein, putative	-5.968	0.00%	0.00%	91.89	15.397
TcCLB.419169.20	ribosomal RNA large subunit beta, 5' partial	∞	0.00%	0.00%	91.63	0
TcCLB.509463.41	retrotransposon hot spot (RHS) protein, putative	∞	0.00%	0.00%	91.53	0
TcCLB.509033.60	hypothetical protein, conserved	∞	0.25%	0.00%	90.12	0
TcCLB.509259.126	hypothetical protein	∞	0.00%	0.00%	90.05	0

TcCLB.509911.30	hypothetical protein	-4.651	0.00%	0.00%	89.11	19.162
TcCLB.505939.10	hypothetical protein	∞	0.03%	0.00%	89.00	0
TcCLB.508799.250	hypothetical protein, conserved	-5.627	0.00%	0.00%	86.05	15.293
TcCLB.510687.149	coatomer alpha subunit, putative	∞	0.00%	0.00%	86.01	0
TcCLB.503943.10	putative	-4.023	0.00%	0.00%	85.28	21.199
TcCLB.511509.30	protein kinase, putative	-4.089	0.00%	0.00%	83.07	20.315
TcCLB.511285.120	putative	-9.046	0.00%	0.00%	81.45	9.004
TcCLB.506959.20	hypothetical protein, conserved	-4.078	0.00%	0.00%	80.17	19.659
TcCLB.511427.19	hypothetical protein, conserved	∞	0.00%	0.00%	76.76	0
TcCLB.511773.30	hypothetical protein	∞	0.00%	0.00%	76.12	0
TcCLB.509165.10	trans-sialidase (pseudogene), putative	-9.543	0.00%	0.00%	75.30	7.891
TcCLB.511771.199	hypothetical protein	∞	0.00%	0.00%	74.80	0
TcCLB.420293.50	trans-sialidase (pseudogene), putative	∞	0.00%	0.00%	73.10	0
TcCLB.504423.15	hypothetical protein, conserved	∞	0.00%	0.00%	72.66	0
TcCLB.507949.4	putative	∞	0.00%	0.00%	72.04	0
TcCLB.510095.80	hypothetical protein	∞	0.00%	0.00%	71.67	0
TcCLB.506355.80	flagellum transition zone component, putative	-4.861	0.00%	0.00%	71.27	14.662
TcCLB.426799.9	hypothetical protein, conserved	∞	0.02%	0.00%	68.67	0
TcCLB.506993.190	hypothetical protein	∞	0.00%	0.00%	68.10	0
TcCLB.511581.20	fatty acyl CoA syntetase 1	∞	0.00%	0.00%	67.84	0
TcCLB.507801.19	dynein heavy chain, putative	∞	0.00%	0.00%	67.74	0
TcCLB.511603.40	hypothetical protein	∞	0.00%	0.00%	66.89	0
TcCLB.503717.15	hypothetical protein	∞	0.03%	0.00%	66.53	0
TcCLB.507887.20	ubiquitin hydrolase, putative,cysteine peptidase, Clan CA, family C19,	-5.895	0.00%	0.00%	66.28	11.243
TcCLB.506529.324	hypothetical protein, conserved	∞	0.00%	0.00%	65.25	0
TcCLB.504085.30	RNA-binding protein, putative	∞	0.49%	0.00%	64.15	0
TcCLB.506925.70	epsin, putative	∞	0.33%	0.00%	63.48	0
TcCLB.506317.20	hypothetical protein, conserved	-6.407	0.00%	0.00%	63.25	9.872
TcCLB.508235.10	hypothetical protein, conserved (pseudogene)	∞	0.24%	0.00%	62.79	0
TcCLB.508797.29	hypothetical protein, conserved	∞	0.01%	0.00%	62.19	0
TcCLB.506245.302	mucin-associated surface protein (MASP), putative	∞	0.36%	0.00%	62.01	0
TcCLB.504123.50	hypothetical protein, conserved (pseudogene)	-6.686	0.00%	0.00%	61.17	9.149
TcCLB.507357.20	hypothetical protein	∞	0.00%	0.00%	60.21	0
TcCLB.503463.30	protein kinase, putative	-9.177	0.12%	0.00%	59.59	6.493
TcCLB.509259.111	N-acetyltransferase complex ARD1 subunit (pseudogene), putative	∞	0.00%	0.00%	59.38	0
TcCLB.511283.210	hypothetical protein, conserved	-5.879	0.01%	0.00%	59.33	10.091
TcCLB.460197.10	cysteine peptidase (pseudogene), putative	∞	0.00%	0.00%	59.12	0
TcCLB.511727.190	hypothetical protein, conserved	∞	0.00%	0.00%	58.68	0
TcCLB.511819.30	hypothetical protein, conserved	∞	0.00%	0.00%	58.47	0
TcCLB.506885.10	glycosyl transferase, putative	-4.851	0.00%	0.00%	58.14	11.986

TcCLB.507053.110	hypothetical protein, conserved	-7.431	0.00%	0.00%	58.00	7.804
TcCLB.511283.194	hypothetical protein, conserved	∞	0.00%	0.00%	56.83	0
TcCLB.503827.94	chitin binding-like protein (pseudogene), putative	∞	0.00%	0.00%	56.38	0
TcCLB.509065.160	eukaryotic translation initiation factor 3 subunit 7-like protein, putative	-4.892	0.55%	0.00%	55.25	11.295
TcCLB.508043.20	metallo-beta-lactamase-like protein, putative	-4.016	0.00%	0.00%	54.71	13.624
TcCLB.511021.99	hypothetical protein, conserved	∞	0.00%	0.00%	53.72	0
TcCLB.508799.140	transcription modulator/accessory protein, putative	∞	0.00%	0.00%	53.18	0
TcCLB.507641.239	hypothetical protein, conserved	∞	0.00%	0.00%	52.43	0
TcCLB.506595.140	hypothetical protein	∞	0.62%	0.00%	52.37	0
TcCLB.438147.9	hypothetical protein, conserved	∞	0.00%	0.00%	52.27	0
TcCLB.504045.5	hypothetical protein, conserved	∞	0.03%	0.00%	51.45	0
TcCLB.511277.130	hypothetical protein, conserved	-4.185	0.00%	0.00%	51.35	12.271
TcCLB.506297.230	hypothetical protein, conserved	-5.258	0.00%	0.00%	51.33	9.762
TcCLB.511803.30	hypothetical protein, conserved	∞	0.00%	0.00%	51.13	0
TcCLB.509317.80	hypothetical protein, conserved	∞	0.00%	0.00%	50.91	0
TcCLB.511365.80	mitochondrial carrier protein, putative	∞	0.00%	0.00%	50.72	0
TcCLB.506337.190	hypothetical protein, conserved	-4.012	0.00%	0.00%	50.66	12.625
TcCLB.506503.50	hypothetical protein, conserved	∞	0.00%	0.00%	50.18	0
TcCLB.508823.120	ribosomal protein S20, putative	∞	0.00%	0.00%	50.16	0
