# Supplementary material for: Eukaryotic translation elongation factor-1 alpha is associated with a specific subset of mRNAs in Trypanosoma cruzi
Source: BMC Microbiol. 2015 May 19;15:104. doi: 10.1186/s12866-015-0436-2 (PMC4436862; doi:10.1186/s12866-015-0436-2)
Supplement: Additional file 2: — RNAs associated to EF-1a in stressed epimastigotes. [file 12866_2015_436_MOESM2_ESM.pdf]

Feature ID	Product	Baggerley's test: STR EF1a vs CT EF1a normalized values - Weighted proportions fold change	Baggerley's test: STR EF1a vs CT EF1a normalized values - Bonferroni	Baggerley's test: STR EF1a vs CT EF1a normalized values - FDR p-value correction	CT EF1a - Normalized means	STR EF1a - Normalized means
TcCLB.508111.30	glutamate dehydrogenase, putative	-15.67	0.00%	0.00%	95.37	1494.80
TcCLB.511391.120	glutamamyl carboxypeptidase, putative,metallo-peptidase, clan MH, family M18, putative	-4.25	0.00%	0.00%	345.20	1467.15
TcCLB.508719.70	hypothetical protein, conserved	-7.59	0.01%	0.00%	97.96	743.37
TcCLB.507715.10	D-alanyl-glycyl endopeptidase-like protein, putative,cysteine peptidase, Clan CA, family C51, putative	-5.08	3.17%	0.00%	146.15	742.74
TcCLB.504105.140	enolase, putative	-6.52	0.00%	0.00%	105.70	688.68
TcCLB.506925.319	isocitrate dehydrogenase, putative	-7.39	0.07%	0.00%	82.34	608.42
TcCLB.511517.37	reticulon domain protein, putative	-5.70	0.00%	0.00%	91.15	519.44
TcCLB.511277.60	alcohol dehydrogenase, putative	-8.92	0.03%	0.00%	57.83	515.52
TcCLB.506621.30	ribonucleoside-diphosphate reductase small chain, putative	-5.82	0.00%	0.00%	84.04	488.99
TcCLB.504191.10	sterol 24-c-methyltransferase, putative	-10.05	0.00%	0.00%	47.34	475.78
TcCLB.504153.280	hypothetical protein, conserved	-8.19	0.00%	0.00%	55.29	453.10
TcCLB.504045.110	urocanate hydratase, putative	-6.48	2.06%	0.00%	67.24	435.84
TcCLB.471709.10	ribosomal RNA large subunit alpha, 3' partial	∞	0.62%	0.00%	0.00	422.02
TcCLB.509353.60	calmodulin, putative	-10.86	0.00%	0.00%	35.96	390.55
TcCLB.509499.14	tryparedoxin peroxidase, putative	-15.58	0.00%	0.00%	25.03	390.03
TcCLB.510293.40	ubiquitin/ribosomal protein S27a, putative	-14.07	0.00%	0.00%	26.80	377.06
TcCLB.510661.250	cystathione gamma lyase, putative	-6.51	0.18%	0.00%	55.56	361.93
TcCLB.508999.260	calpain-like cysteine peptidase (pseudogene), putative	-8.06	0.00%	0.00%	43.37	349.47
TcCLB.436429.10	ribosomal RNA large subunit beta, 3' partial	-19.80	0.00%	0.00%	17.55	347.60
TcCLB.507029.30	heat shock 70 kDa protein, mitochondrial precursor, putative	-4.29	0.00%	0.00%	77.95	334.00
TcCLB.510121.20	hexokinase, putative	-8.04	0.00%	0.00%	36.11	290.16
TcCLB.506789.280	methylthioadenosine phosphorylase, putative	-5.16	0.00%	0.00%	55.34	285.66
TcCLB.506247.190	nuclear lim interactor-interacting factor, putative	∞	3.25%	0.00%	0.00	284.04

TcCLB.510669.40	hypothetical protein	-6.14	0.00%	0.00%	45.82	281.26
TcCLB.506321.290	stress-induced protein sti1, putative	-4.59	0.09%	0.00%	61.20	280.64
TcCLB.510535.100	cysteine peptidase C (CPC), putative,cathepsin B-like protease, putative,CPC cysteine peptidase, clan CA, family C1, putative	-8.12	0.00%	0.00%	34.02	276.30
TcCLB.509837.10	hypothetical protein, conserved	-9.33	0.53%	0.00%	28.06	261.84
TcCLB.508099.40	hypothetical protein	∞	0.00%	0.00%	0.00	259.21
TcCLB.503975.50	hypothetical protein, conserved	-4.76	0.00%	0.00%	52.04	247.97
TcCLB.509793.10	kinetoplast DNA-associated protein, putative	∞	4.11%	0.00%	0.00	237.57
TcCLB.509569.100	nucleoside phosphorylase, putative	-7.20	0.96%	0.00%	32.94	237.00
TcCLB.509395.100	cytochrome b-domain protein, putative	-11.52	0.00%	0.00%	19.73	227.27
TcCLB.460197.10	cysteine peptidase (pseudogene), putative	∞	0.00%	0.00%	0.00	216.37
TcCLB.511583.40	tricarboxylate carrier, putative	-6.09	0.00%	0.00%	34.64	210.97
TcCLB.507681.20	succinyl-CoA ligase [GDP-forming] beta-chain, putative	-5.17	0.00%	0.00%	40.68	210.38
TcCLB.503939.110	hypothetical protein, conserved	-7.46	1.03%	0.00%	27.18	202.72
TcCLB.510535.10	hypothetical protein, conserved	-26.44	0.00%	0.00%	7.62	201.40
TcCLB.509791.189	hypothetical protein, conserved	-7.43	0.00%	0.00%	26.67	198.13
TcCLB.509917.19	retrotransposon hot spot (RHS) protein, putative	-4.19	0.00%	0.00%	46.95	196.60
TcCLB.509239.10	fatty acid desaturase, putative	-7.49	0.00%	0.00%	26.18	196.14
TcCLB.509601.140	hypothetical protein	∞	0.00%	0.00%	0.00	189.63
TcCLB.503975.40	hypothetical protein, conserved	-13.34	0.03%	0.00%	14.08	187.87
TcCLB.507305.20	casein kinase, putative	∞	0.00%	0.00%	0.00	186.93
TcCLB.503833.50	hypothetical protein, conserved	-5.49	0.00%	0.00%	33.62	184.69
TcCLB.508971.40	acetylornithine deacetylase-like, putative	-12.78	0.00%	0.00%	14.35	183.36
TcCLB.506529.508	glucose-6-phosphate isomerase, glycosomal, putative	-4.86	0.00%	0.00%	37.32	181.47
TcCLB.509167.140	hypothetical protein, conserved	-12.05	0.00%	0.00%	14.87	179.26
TcCLB.510795.10	tyrosine aminotransferase	-4.12	1.31%	0.00%	40.87	168.60
TcCLB.510301.20	fructose-bisphosphate aldolase, glycosomal, putative	∞	0.00%	0.00%	0.00	165.88
TcCLB.511239.49	adenosine 5'-monophosphoramidase, putative	∞	0.00%	0.00%	0.00	163.74
TcCLB.507951.299	hypothetical protein, conserved	-10.28	2.57%	0.00%	15.84	162.76
TcCLB.507787.100	gamma-glutamylcysteine synthetase, putative	-9.87	0.01%	0.00%	16.37	161.63
TcCLB.508301.20	mitochondrial processing peptidase, beta subunit, putative,metallo-peptidase, Clan ME, Family M16, putative	∞	1.19%	0.00%	0.00	151.95
TcCLB.507165.30	hypothetical protein, conserved	∞	4.11%	0.00%	0.00	146.20
TcCLB.510121.50	calmodulin, putative	-4.72	0.00%	0.00%	30.38	143.48
TcCLB.506241.170	40S ribosomal protein S6, putative	∞	1.50%	0.00%	0.00	139.62
TcCLB.509233.180	ATPase beta subunit, putative	-4.23	1.01%	0.00%	32.84	138.84

TcCLB.511909.40	succinate dehydrogenase flavoprotein, putative	-14.89	0.48%	0.00%	9.31	138.73
TcCLB.510797.10	2-oxoglutarate dehydrogenase subunit, putative	-4.87	0.00%	0.00%	28.18	137.35
TcCLB.508505.4	hypothetical protein, conserved	∞	0.00%	0.00%	0.00	134.37
TcCLB.508409.180	hypothetical protein, conserved	-7.53	0.00%	0.00%	17.22	129.56
TcCLB.511801.60	mitochondrial DEAD box protein, putative	-4.09	0.00%	0.00%	31.33	128.22
TcCLB.506977.90	proteasome regulatory non-ATPase subunit 5, putative,19S proteasome regulatory subunit, putative	-5.07	0.00%	0.00%	23.62	119.70
TcCLB.506441.20	hypothetical protein, conserved	-7.35	0.01%	0.00%	15.74	115.73
TcCLB.503587.80	mucin-associated surface protein (MASP, pseudogene), putative	∞	0.00%	0.00%	0.00	115.39
TcCLB.506247.340	hypothetical protein, conserved	∞	0.00%	0.00%	0.00	115.10
TcCLB.511635.40	protein tyrosine phosphatase, putative	-4.72	0.00%	0.00%	24.18	114.17
TcCLB.506801.20	mucin-like glycoprotein, putative	-5.04	3.21%	0.00%	22.50	113.35
TcCLB.504109.90	ubiquitin carboxyl-terminal hydrolase, putative,cysteine peptidase, Clan CA, family C12, putative	-4.72	0.00%	0.00%	23.97	113.21
TcCLB.511907.310	hypothetical protein, conserved	-5.38	0.00%	0.00%	20.51	110.38
TcCLB.508859.40	leucine-rich repeat protein (LRRP), putative	∞	0.00%	0.00%	0.00	110.05
TcCLB.503837.40	hypothetical protein, conserved	∞	0.00%	0.00%	0.00	107.64
TcCLB.504005.54	hypothetical protein, conserved	∞	0.00%	0.00%	0.00	107.64
TcCLB.511909.60	hypothetical protein, conserved	∞	0.00%	0.00%	0.00	106.93
TcCLB.507765.120	hypothetical protein, conserved	∞	0.00%	0.00%	0.00	105.46
TcCLB.508043.20	metallo-beta-lactamase-like protein, putative	-7.43	0.00%	0.00%	13.62	101.20
TcCLB.510421.250	hypothetical protein, conserved	-6.67	0.65%	0.00%	15.03	100.27
TcCLB.511277.400	UDP-galactose transporter, putative	∞	0.00%	0.00%	0.00	97.31
TcCLB.508643.20	hypothetical protein, conserved	∞	0.00%	0.00%	0.00	94.79
TcCLB.504105.20	eukaryotic translation initiation factor 5, putative	∞	1.50%	0.00%	0.00	92.22
TcCLB.509937.50	hypothetical protein, conserved	-4.47	0.16%	0.00%	20.56	91.91
TcCLB.509029.80	hypothetical protein	-4.36	0.00%	0.00%	20.93	91.21
TcCLB.506715.40	hypothetical protein, conserved	∞	0.07%	0.00%	0.00	91.10
TcCLB.508719.60	kinetoplast DNA-associated protein, putative	∞	0.00%	0.00%	0.00	90.82
TcCLB.506263.30	D-isomer specific 2-hydroxyacid dehydrogenase-protein, putative	-5.51	3.47%	0.00%	16.37	90.19
TcCLB.507951.270	hypothetical protein, conserved	-7.55	0.00%	0.00%	11.84	89.42
TcCLB.511575.30	hypothetical protein, conserved	∞	0.00%	0.00%	0.00	88.31
TcCLB.506417.70	hypothetical protein	-4.09	0.00%	0.00%	20.58	84.24
TcCLB.506725.20	hypothetical protein, conserved	-4.36	0.00%	0.00%	19.32	84.23
TcCLB.506469.130	hypothetical protein	∞	0.00%	0.00%	0.00	82.16
TcCLB.510943.50	delta-1-pyrroline-5-carboxylate dehydrogenase, putative	-8.09	0.00%	0.00%	10.11	81.76

TcCLB.506727.100	enoyl-CoA hydratase/isomerase family protein, putative	∞	0.00%	0.00%	0.00	81.30
TcCLB.507093.260	ABC transporter, putative	-9.38	0.06%	0.00%	8.58	80.47
TcCLB.509331.180	aldose 1-epimerase-like protein, putative	-5.38	0.00%	0.00%	14.91	80.25
TcCLB.510101.50	lanosterol 14-alpha-demethylase, putative	∞	0.00%	0.00%	0.00	78.64
TcCLB.506401.12	protein kinase domain, putative	∞	4.11%	0.00%	0.00	77.57
TcCLB.509599.130	serine/threonine protein kinase, putative	∞	0.00%	0.00%	0.00	77.53
TcCLB.507275.40	hypothetical protein, conserved	∞	4.11%	0.00%	0.00	76.79
TcCLB.510339.80	hypothetical protein, conserved	∞	4.11%	0.00%	0.00	75.65
TcCLB.511165.109	hypothetical protein, conserved	∞	0.00%	0.00%	0.00	75.33
TcCLB.509455.114	hypothetical protein, conserved	∞	0.00%	0.00%	0.00	75.20
TcCLB.510027.20	transporter, putative	∞	0.00%	0.00%	0.00	75.05
TcCLB.510879.130	hypothetical protein, conserved	-5.48	0.72%	0.00%	13.59	74.47
TcCLB.510439.61	heat shock 70 kDa protein, putative	-4.72	0.00%	0.00%	15.40	72.71
TcCLB.506853.50	2-oxoisovalerate dehydrogenase alpha subunit, putative	∞	0.00%	0.00%	0.00	70.78
TcCLB.503745.30	ascorbate-dependent peroxidase, putative	∞	0.00%	0.00%	0.00	70.67
TcCLB.511517.130	hypothetical protein, conserved	∞	0.00%	0.00%	0.00	70.46
TcCLB.503555.30	trypanothione reductase, putative	∞	0.00%	0.00%	0.00	69.62
TcCLB.506925.20	hypothetical protein, conserved	-4.09	0.00%	0.00%	17.01	69.61
TcCLB.510943.90	hypothetical protein, conserved	∞	0.00%	0.00%	0.00	68.38
TcCLB.511531.30	hypothetical protein, conserved	-6.80	0.00%	0.00%	10.06	68.36
TcCLB.504149.150	hypothetical protein, conserved	-4.75	0.10%	0.00%	14.38	68.35
TcCLB.508045.70	hypothetical protein, conserved	∞	0.00%	0.00%	0.00	68.33
TcCLB.511421.100	hypothetical protein, conserved	∞	0.00%	0.00%	0.00	68.10
TcCLB.504037.10	hypothetical protein, conserved	∞	0.00%	0.00%	0.00	67.59
TcCLB.506945.250	hypothetical protein, conserved	∞	2.00%	0.00%	0.00	66.55
TcCLB.503525.4	hypothetical protein, conserved	∞	0.00%	0.00%	0.00	64.10
TcCLB.508909.310	hypothetical protein	∞	0.00%	0.00%	0.00	61.53
TcCLB.504163.60	hypothetical protein, conserved	∞	0.00%	0.00%	0.00	61.19
TcCLB.506363.80	aminoacylase, putative,N-acyl-L-amino acid amidohydrolase, putative	∞	0.00%	0.00%	0.00	61.19
TcCLB.508719.30	hypothetical protein, conserved	∞	0.00%	0.00%	0.00	60.24
TcCLB.507089.170	hypothetical protein, conserved	∞	0.00%	0.00%	0.00	58.09
TcCLB.511807.110	GMP synthase, putative,glutamine amidotransferase, putative	-6.67	1.11%	0.00%	8.63	57.60
TcCLB.457251.10	3-oxo-5-alpha-steroid 4-dehydrogenase, putative	∞	4.11%	0.00%	0.00	57.59
TcCLB.506789.200	ubiquitin-conjugating enzyme, putative	-4.09	0.00%	0.00%	13.82	56.57
TcCLB.506457.10	methionine aminopeptidase 2, putative,metallo-peptidase, clan MG, family M24, putative	-4.72	0.00%	0.00%	11.94	56.37
TcCLB.511269.50	protein kinase A catalytic subunit, putative	∞	0.00%	0.00%	0.00	56.07
TcCLB.504643.49	hypothetical protein, conserved	∞	2.00%	0.00%	0.00	54.77

TcCLB.510295.59	hypothetical protein, conserved	∞	0.00%	0.00%	0.00	54.41
TcCLB.453917.9	hypothetical protein, conserved	∞	0.00%	0.00%	0.00	52.45
TcCLB.506735.80	hypothetical protein, conserved	∞	0.00%	0.00%	0.00	52.35
TcCLB.511439.70	hypothetical protein, conserved	∞	0.00%	0.00%	0.00	50.66
TcCLB.509353.20	hypothetical protein, conserved	∞	4.11%	0.00%	0.00	50.18
