# Supplementary material for: Eukaryotic translation elongation factor-1 alpha is associated with a specific subset of mRNAs in Trypanosoma cruzi
Source: BMC Microbiol. 2015 May 19;15:104. doi: 10.1186/s12866-015-0436-2 (PMC4436862; doi:10.1186/s12866-015-0436-2)
Supplement: Additional file 3: — Supplemental methods. [file 12866_2015_436_MOESM3_ESM.docx]

**Supplemental material**

**Quantitative PCR**

The transcripts associated to EF-1 alpha in epimastigotes an epimastigotes under nutritional stress were immunoprecipitated as for the RNA-seq experiment (see EF-1a immunoprecipitation RNA-seq methods) and submitted to RNA extraction with RNeasy™ kit (Qiagen). As a control we used the pre-immune serum under the same conditions. The RNAs were reverse transcribed with the ImProm-II™ Reverse Transcriptase kit (Promega) using 10 µM of random primer for 2 h at 42°C. qPCR reactions were performed with SYBR® Green PCR Master Mix (Applied Biosystems) using 1 uL of cDNA/20 µL reaction and 5 µM of specific forward and reverse primers for Heat shock protein 100, Receptor for activated C kinase, D-alanyl-glycyl endopeptidase-like protein, Enolase and Glyceraldehyde 3-phosphate dehydrogenase (Table S3). Program setup was as follows in the Lightcycler 96 (Roche): initial denaturation at 95°C for 15 min and 50 cycles of 95°C for 15 sec, 60 °C for 20 sec and 72°C for 45 sec. Data Analysis were performed in biological triplicates using Pfaffl model [1], where the reference used was GAPDH amplification data.

**Supplemental Figure 1** – Controls of the Immunofluorescence assay.

In the top panel the primary antibody used was the pre-immune serum diluted 1:300, the secondary antibody used was Alexa-488 goat anti-rabbit (1:400). The bottom panel is the control using only the secondary antibody diluted 1:400. Kinetoplasts and nuclei were stained with DAPI.

**Supplemental Figure 2** – Immunoprecipitation western blot.

Epimastigote protein extract was used for the immunoprecipitation with the antibody against EF-1α as described in the methods section. The elution was performed using SDS-Page loading buffer (62.5 mM Tris-HCl pH 6.8, 2.5% SDS, 5% β-mercaptoethanol, 10% glycerol and 0.002% bromophenol blue) at 95 ^o^C for 5 min. The input and the elution antibody dilution used was 1:100 for the western blot, the IgG chains and the EF-1α protein are indicated by arrows and the sizes indicated in kDa.

**Supplemental figure 3** – Validation of the RNA-seq targets by qRT-PCR.

The experiment was performed in biological quadruplicates. Error bar indicates standard error between biological and technical replicates. The ratio indicated was calculated comparing the Epimastigote/control relative expression or Stress/control relative expression.

**Reference:**

1 - Pfaffl MW (2001) **A new mathematical model for relative quantification in real-time RT-PCR**. *Nucleic Acids Research* 2001, **29**(9): 2002–2007. doi: 10.1093/nar/29.9.e45
