# Supplementary figures and images for: Eukaryotic translation elongation factor-1 alpha is associated with a specific subset of mRNAs in Trypanosoma cruzi
Source: BMC Microbiol. 2015 May 19;15:104. doi: 10.1186/s12866-015-0436-2 (PMC4436862; doi:10.1186/s12866-015-0436-2)

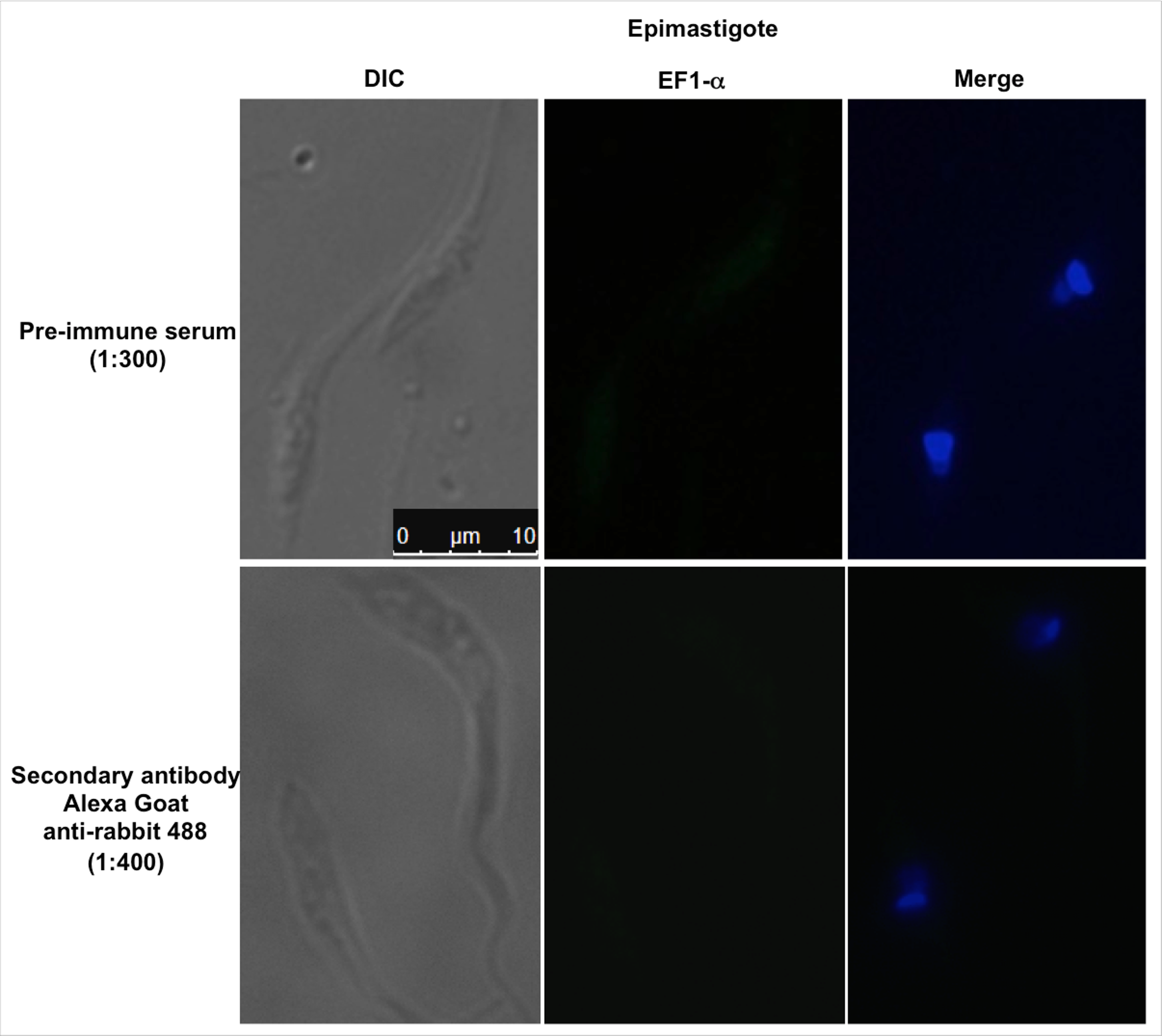

Supplement: Additional file 4: — Controls of the immunofluorescence assay. [file 12866_2015_436_MOESM4_ESM.tiff]

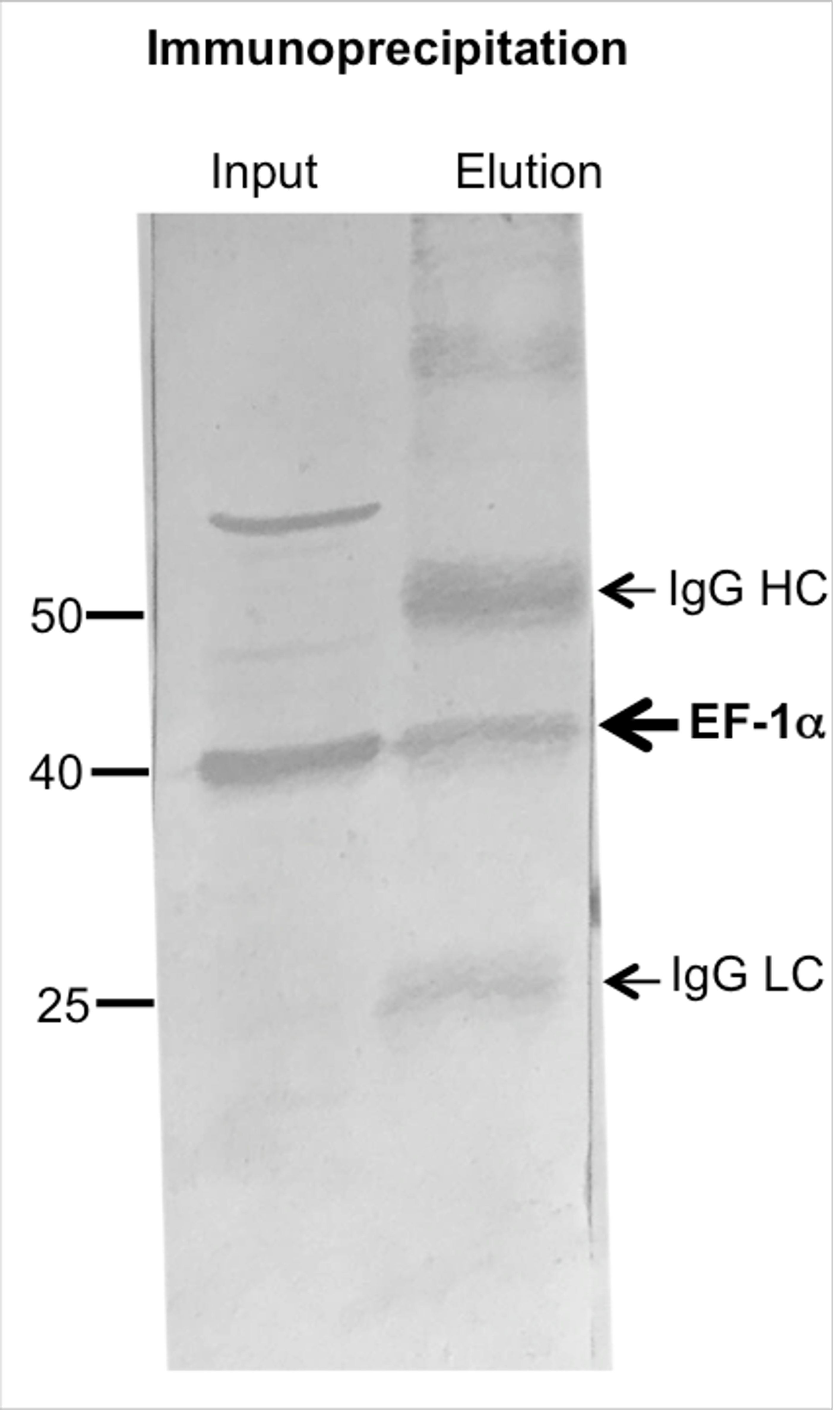

Supplement: Additional file 5: — EF-1a immunoprecipitation western-blot. [file 12866_2015_436_MOESM5_ESM.tiff]

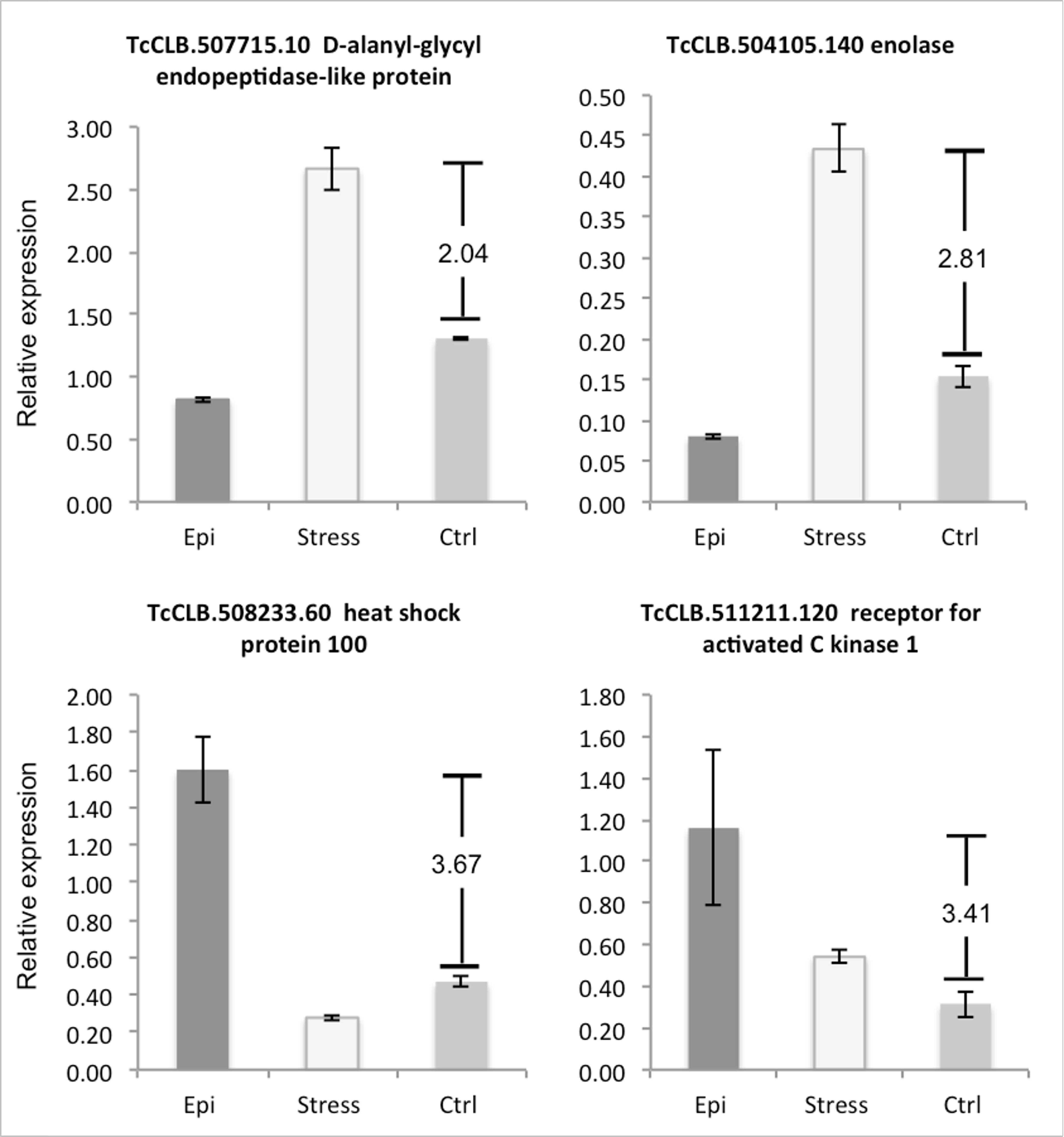

Supplement: Additional file 6: — Validation of the RNA-seq by qRT-PCR. [file 12866_2015_436_MOESM6_ESM.tiff]
